# Study on Freeze–Thaw Cycle Performance and Regional Service Life Prediction of Hydrophobic Aerogel-Modified ACEPS Boards

**DOI:** 10.3390/ma18112646

**Published:** 2025-06-05

**Authors:** Lu Lu, Rongyu Chen, Mingming Wang, Wenjia Xi, Shan Yun, Haodong Wang

**Affiliations:** 1 Jiangsu Provincial Engineering Laboratory for Advanced Materials of Salt Chemical Industry, Huaiyin Institute of Technology, Huai’an 223003, China; yunshan116@126.com (S.Y.);; 2 Jiangsu Smart Factory Engineering Research Center, Huaiyin Institute of Technology, Huai’an 223003, China; 3 Faculty of Architectural Engineering, Huaiyin Institute of Technology, Huai’an 223201, China; 4 College of Water Resources and Environmental Engineering, Zhejiang University of Water Resources and Electric Power, Hangzhou 310018, China

**Keywords:** hydrophobic aerogel, freeze–thaw cycles, external wall insulation materials, freeze–thaw resistance, service life prediction

## Abstract

The aim of this study is to systematically investigate the influence of hydrophobic aerogel on the performance of aerogel cement-based expanded polystyrene (EPS) insulation board (ACEPS board) under freeze–thaw cycles (FTCs) and to predict its service life in four typical climate zones: Beijing, Harbin, Urumqi, and Nanjing. The effects of aerogel content on compressive strength, volumetric water absorption, thermal conductivity, and pore structure evolution of ACEPS were thoroughly analyzed through FTC testing. The results demonstrated that aerogel significantly reduced the volumetric water absorption of ACEPS due to its excellent hydrophobicity, thereby decreasing the compressive strength attenuation from 40% to 24%, suppressing the increase in thermal conductivity from 0.0130 to 0.0055 W/(m·K), and mitigating pore structure degradation. In the regional service life prediction, aerogel-modified ACEPS exhibited significantly improved freeze–thaw resistance in the cold climates of Harbin and Urumqi, as well as in the high freeze–thaw frequency environment of Beijing. Notably, specimens with high aerogel content demonstrated outstanding structural and functional durability. This study provides a theoretical foundation and practical guidance for incorporating aerogel in the optimized designs and applications of thermal insulation building materials in cold regions.

## 1. Introduction

The thermal performance of a building envelope plays an important role in controlling building energy consumption and regulating the indoor thermal environment. Heat transfer losses through the envelope account for approximately 30% to 50% of the total energy consumption of the building, with external wall heat loss being particularly significant [1,2]. To meet the energy-saving targets outlined in Chinese standards GB 55015-2021 [3] and GB/T 51350-2019 [4], modern building design is increasingly focused on optimizing envelope thermal performance to satisfy the technical requirements of near-zero energy buildings in China [5,6]. Heating energy consumption in cold regions is notably higher than that in other climate zones due to persistently low winter temperatures. In response, underfloor heating systems are widely adopted for their efficient radiant heat transfer. However, the thermal stability of external walls is critical for minimizing heat loss during heating and enhancing the energy efficiency of these systems [7,8]. Therefore, exterior wall insulation materials must exhibit excellent thermal performance, weather resistance, and long-term durability to maintain low thermal conductivity under prolonged service conditions and fulfill building energy efficiency demands.

Building envelopes in cold regions are continuously exposed to complex environmental stresses such as freeze–thaw cycles (FTCs). FTCs damage the microstructure of materials through pore water freezing and expansion stress, resulting in the degradation of mechanical properties and the deterioration of thermal performance [9,10]. Investigating the long-term stability of thermal insulation materials in cold environments is essential for improving building energy efficiency and extending their service life. Niu et al. [11] report that extruded polystyrene (XPS) boards meet the durability requirements for cold regions, although their compressive strength reduces after 400 FTCs. Jiang et al. [12] observed that thicker XPS boards exhibited higher compressive strength under the same number of FTCs. Chen et al. [13] and Wu et al. [14] demonstrated that high-density foam concrete and fly ash-based foam concrete retained some freeze–thaw stability after repeated FTCs, though strength degradation increased with more cycles. Material modification has proven effective in enhancing freeze–thaw resistance. He et al. [15] reduced compressive strength loss to 8.1% by incorporating microbeads into foam concrete. Gencel et al. [16] reported that the addition of glass sand significantly reduced the rate of strength loss in foam concrete. Moreover, Zhang et al. [17] indicated that applied loads could mitigate performance degradation in foam concrete.

Volumetric water absorption and thermal conductivity are critical indicators of insulation performance, with their variations significantly affecting both insulation efficiency and durability [18,19,20,21]. Under FTCs, air within pores is gradually replaced by water, and thermal conductivity increases due to the high conductivity of water and ice (0.60 and 2.2 W/(m·K), respectively) [22,23,24]. Li et al. [25] found that the closed-pore structure of polyphenolic insulation materials was destroyed under FTCs, leading to increased water absorption and thermal conductivity. Zhang et al. [26] and Ming et al. [27] reported that after 300 FTCs, the thermal conductivity of unimmersed polyurethane foam specimens increased by less than 5%, whereas that of immersed specimens nearly doubled, indicating a strong correlation between water absorption and thermal conductivity deterioration. Han et al. [28] further investigated thermal conductivity changes in various insulation materials under water absorption and freezing. Their findings showed that the thermal conductivity of polyphenol, polyurethane, and polystyrene foams increased by factors of 11.52, 3.00, and 0.67, respectively, in the thawed state after maximum water absorption. These results suggest that the cumulative effect of water in the frozen state is the primary driver of thermal conductivity degradation.

In summary, FTCs have a significant impact on the mechanical and thermal properties of thermal insulation materials, with their effects closely tied to material microstructure and water content. Optimizing material structure and employing appropriate modifications are key strategies for improving long-term performance.

Aerogel insulation materials have become a research focus in the field of building energy efficiency in recent years due to their extremely low thermal conductivity and excellent thermal insulation performance [29,30]. To address the limitations of low mechanical strength and high cost, aerogel is commonly compounded with traditional insulation materials to improve its thermal insulation performance [31,32,33,34,35,36,37,38,39]. The behavior of aerogel materials under humid and freeze–thaw conditions is critical for their practical applications. Lakatos et al. [40] reported that while the thermal conductivity of dry aerogels remained stable over extended FTCs, wet aerogels exhibited a marked increase in thermal conductivity due to higher water content, indicating that freeze–thaw degradation primarily results from water-induced structural damage. Nosrati and Berardi [41] further examined the performance of aerogel-reinforced materials under extremely hot and humid conditions and found that aerogel gypsum boards were particularly sensitive to high humidity, with thermal conductivity increasing by 100% at 95% relative humidity, whereas temperature had a comparatively minor effect. These findings highlight the dominant role of humidity in the degradation of the aerogel composite’s properties. The performance of aerogel insulation materials under complex environmental conditions is, therefore, significantly influenced by humidity and water content, offering important insights for the design and application of aerogel composites in harsh environments.

Although CEPS boards exhibit high thermal insulation and flame retardancy, the relatively high thermal conductivity of cement limits their ability to meet stricter energy efficiency standards. Lu et al. [42] proposed a hydrophobic nanosilica aerogel dispersion method for cementitious composites and successfully developed a novel type of non-combustible exterior wall insulation board (ACEPS board), by constructing a composite system using cement as the inorganic binder, EPS as the coarse thermal insulation aggregate, and aerogel as the fine thermal insulation aggregate. This material improved both flame resistance and thermal insulation performance, offering a new direction for exterior wall insulation design. However, the influence of hydrophobic aerogel on the freeze–thaw resistance of CEPS boards remains underexplored.

Therefore, this study comprehensively investigated the changes in compressive strength, volumetric water absorption, thermal conductivity, and pore structure of ACEPS boards after FTC exposure to reveal their freeze–thaw behavior from multiple perspectives. Furthermore, to assess the long-term service life of ACEPS boards under varying climatic conditions, four representative regions—Beijing, Harbin, Urumqi, and Nanjing—were selected for quantitative analysis of FTC-induced degradation based on local environmental conditions. This study provides a valuable experimental foundation for the engineering application of ACEPS boards in cold regions and serves as a scientific reference for the optimized design and performance enhancement of thermal insulation materials.

## 2. Materials and Methods

### 2.1. Experimental and Analytical Procedures

ACEPS was selected as the subject of this study, and the preparation process and selection of main materials are detailed in reference [42]. ACEPS specimens with dimensions of 100 mm × 100 mm × 50 mm and 300 mm × 300 mm × 50 mm were prepared through cutting and machining to accommodate different testing requirements. The experiments were designed for three aerogel-to-cement mass ratios—0, 3, and 5 wt%—with corresponding specimen labels of #1 (0 wt% (control)), #2 (3 wt%), and #3 (5 wt%), respectively. Hanif et al. [36] report that when the aerogel content reaches 5 wt%, the particles begin to agglomerate, compromising the stability of material performance. Accordingly, in our study, we designated 5 wt% as the upper limit for aerogel addition and selected 3 wt% as the intermediate content to evaluate its modification effects. Aerogel is introduced as an additional component (calculated as wt% of the total mass) rather than as a partial replacement for cement. This approach enhances the functional properties of the material while maintaining a constant cement content, thereby ensuring the consistency of the baseline matrix composition. In this study, the characteristics of ACEPS without aerogel (0 wt%)—a modified thermosetting polystyrene foam insulation material—were compared with those of ACEPS containing 3 and 5 wt% aerogel. The comparison results systematically revealed the influence of aerogel incorporation on the freeze–thaw resistance of ACEPS insulation boards. ACEPS specimens with varying aerogel contents were then tested for fundamental properties, including thermal conductivity, compressive strength, volumetric water absorption, and pore structure; the latter was further analyzed in terms of porosity and pore size distribution.

Subsequently, the FTC tests were performed to simulate the material property changes under different FTC counts (10, 30, 50, 100, 150, 200, and 300 cycles), focusing on the degradation patterns of thermal conductivity, compressive strength, volumetric water absorption, and pore structure. Based on the experimental data, a service life prediction model under FTC conditions was developed using the degradation trends of compressive strength and thermal conductivity. The test conditions are summarized in Supplementary Materials. The model evaluates two aspects: structural life (based on compressive strength) and functional life (based on thermal conductivity). Finally, the freeze–thaw service life of ACEPS boards in four representative regions—Beijing, Harbin, Urumqi, and Nanjing—was predicted by constructing an equivalent indoor–outdoor FTC model and incorporating actual climatic data.

### 2.2. Freeze–Thaw Test Program

**Figure 1:**
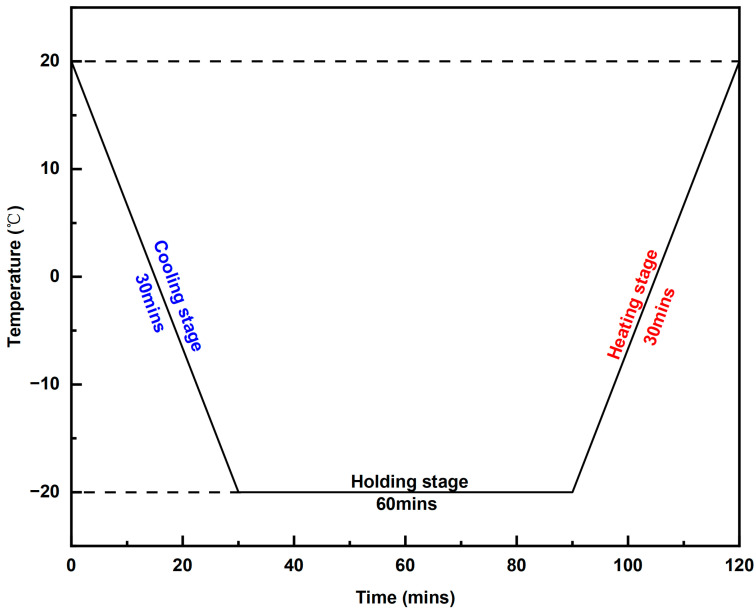
Test cycle for a single FTC.

Our experimental protocol strictly follows the requirements of GB/T 33011-2016 [43], which designates volumetric water absorption and compressive strength retention as the principal evaluation indicators for freeze–thaw resistance in insulation systems. The specimens were first immersed in a water tank for no less than 96 h to ensure complete saturation. After immersion, the specimens were removed, air-dried for 10 min, and gently wiped with a sponge to remove any surface moisture. The prepared specimens were then placed on the shelves of a constant temperature and humidity chamber to begin the FTC test. As illustrated in Figure 1, each complete FTC consists of a cooling phase, a holding phase, and a heating phase. Specifically, the chamber temperature was lowered from 20 to −20 °C within 30 min and maintained at −20 °C for 60 min during the holding phase. Subsequently, the temperature was gradually raised back to 20 °C within 30 min, completing one FTC cycle.

### 2.3. Volumetric Water Absorption

First, the dried specimens were accurately measured for mass and volume. To ensure complete water contact during immersion, wire mesh was used to fix the specimen in place, preventing it from floating due to buoyancy. The vessel was then slowly filled with water until the water level was 25 mm above the upper surface of the specimen, ensuring full submersion. After the specified immersion time, the specimen was removed and left to dry for 10 min in a room-temperature environment. A sponge was then used to gently wipe off any residual surface moisture, preventing excess moisture from affecting the measurement results. Finally, the mass of the specimen was determined using a high-precision balance. Volumetric water absorption was calculated using Equation (1):

(1) $$Wv=\frac{G_{1}-G_{0}}{\rho V}$$

where *W_v_* denotes the volumetric water absorption rate of the specimen; *G*_1_ denotes the mass of the specimen after water absorption; *G*_0_ denotes the mass of the specimen after drying; *ρ* denotes the density of water, and *V* denotes the volume of the specimen after drying. The test results were obtained by averaging the measurements from three specimens to enhance the reliability and accuracy of the data.

### 2.4. Testing Method for Compressive Strength

The compressive test was conducted in accordance with the relevant provisions of the Chinese national standard GB/T 8813-2020 [44]. Prior to the test, standard specimens with dimensions of 100 mm × 100 mm × 50 mm were prepared, ensuring that the surfaces of the specimens were flat and free of significant defects to guarantee the accuracy and repeatability of the results. The compressive test was performed using the FBS-10KNZXP universal tensile testing machine (Xiamen Fobus Instrument Technology Co., Ltd., Xiamen, China) with a constant loading rate of 5 mm/min. During the test, the specimen was continuously compressed under increasing force until yielding occurred or 10% deformation was reached.

### 2.5. Testing Method for Thermal Conductivity

In accordance with the Chinese specification GB/T 10295-2008 [45], specimens measuring 30 cm × 30 cm × 3 cm were tested for thermal conductivity using the GYDR-3030 intelligent thermal conductivity tester (Tianjin Gangyuan Testing Instrument Factory, Tianjin, China). The test was conducted at an average temperature of (25 ± 2) °C, and the result was taken as the arithmetic mean of the measurements from two specimens, with an accuracy of 0.001 W/(m·K).

### 2.6. Characterization Methods for Pore Structure

The pore structure parameters and pore size distribution of ACEPS were analyzed using the AutoPore IV-9500 high-performance automated mercury porosimeter (Mack Instruments, Sandusky, OH, USA). During testing, it was assumed that all pores within the material had an ideal cylindrical tube structure. Changes in mercury intrusion volume were recorded by gradually increasing the intrusion pressure (ranging from 0 to 61,000 psi), which allowed for the characterization of the distribution of pore sizes.

### 2.7. Lifetime Prediction of FTCs Under Natural Conditions

To predict the number of FTCs under natural environmental conditions, the number of FTCs under indoor experimental conditions was converted using the equivalence coefficients for indoor and outdoor FTCs proposed by Chen and Qiao [46]. This conversion enabled the estimation of the freeze–thaw time, T (in years), of the material in the natural environment, as detailed in Equation (2):

(2) $$T=C_{Equivalent}\frac{N_{Indoor}}{N_{Annual}}$$

where *C_Equivalent_* denotes the equivalence factor between the number of laboratory FTCs and the number of outdoor FTCs, which is set to 6.5 in this study. *N_Indoor_* denotes the number of FTCs of the specimen in the laboratory. *N_Annual_* denotes the number of FTCs per year under natural conditions.

## 3. Results and Discussion

### 3.1. Water Absorption in the Natural State

**Figure 2:**
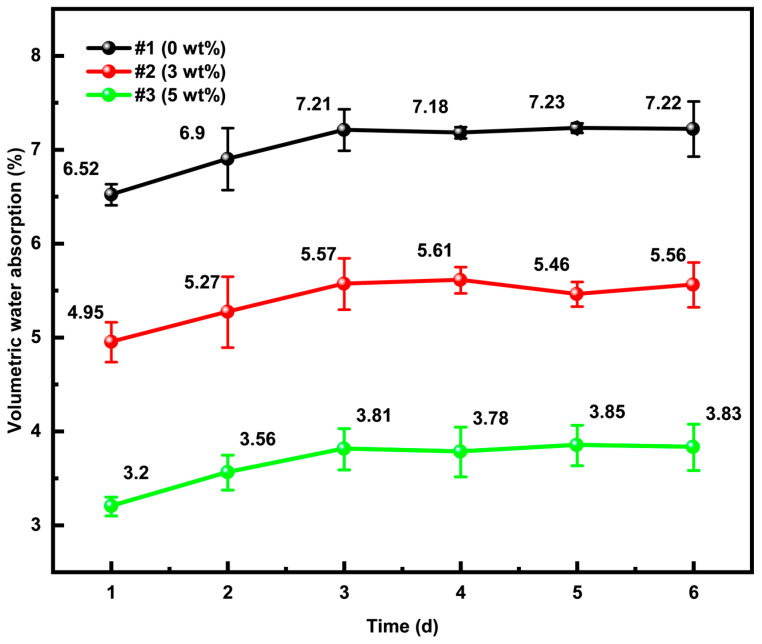
Volumetric water absorption rate of ACEPS in its natural state.

Figure 2 illustrates the variation in volumetric water absorption of ACEPS with immersion time under natural conditions. The results indicate that volumetric water absorption increased significantly with prolonged soaking time. After 1 day of immersion, the volumetric water absorptions for specimens with different aerogel contents were 6.52% (0 wt%), 4.95% (3 wt%), and 3.19% (5 wt%), respectively. After 3 days of immersion, these values increased to 7.21% (0 wt%), 5.57% (3 wt%), and 3.81% (5 wt%), respectively. These findings clearly demonstrate that the volumetric water absorption decreases significantly with increasing aerogel content, highlighting the aerogel’s effective resistance to water penetration. The observed minor changes in the volumetric water absorption rate of the specimen in its natural state on the third, fourth, and fifth days indicate that the maximum rate is achieved on the third day. Therefore, volumetric water absorption rate tests were conducted after subsequent FTCs, using the rate recorded on the third day as the reference.

**Figure 3:**
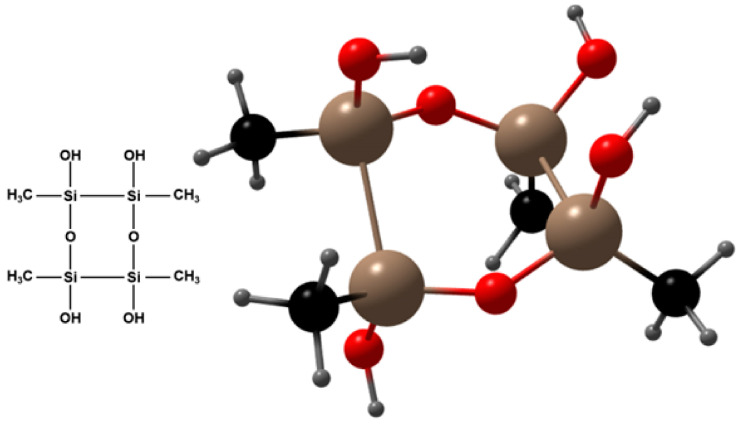
Molecular structure of polysilsesquioxane aerogel.

The chemical structure of the polysilsesquioxane aerogel used in this study [47] can be represented as (CH_3_SiO_1.5_)_n_, where some of the oxygen atoms in the silica molecule are replaced by methyl (-CH_3_) groups. The molecular structure of this aerogel, shown in Figure 3, reveals that its surface is enriched with hydrophobic -CH_3_ groups. The uniform distribution of these hydrophobic groups creates a barrier-like structure within the material, effectively reducing water penetration and enhancing the material’s resistance to water absorption. Additional tests extending the immersion time showed that the volumetric water absorption stabilized after 3 days, presenting a clear upper limit. In comparison to the data from day 3, the volumetric water absorption values on days 4, 5, and 6 fluctuated slightly but did not show significant increases, indicating that the specimens reached maximum water saturation by day 3. This behavior further confirms the effectiveness of the barrier structure formed by the uniform distribution of aerogel in the material.

### 3.2. Volumetric Water Absorption After FTCs

**Figure 4:**
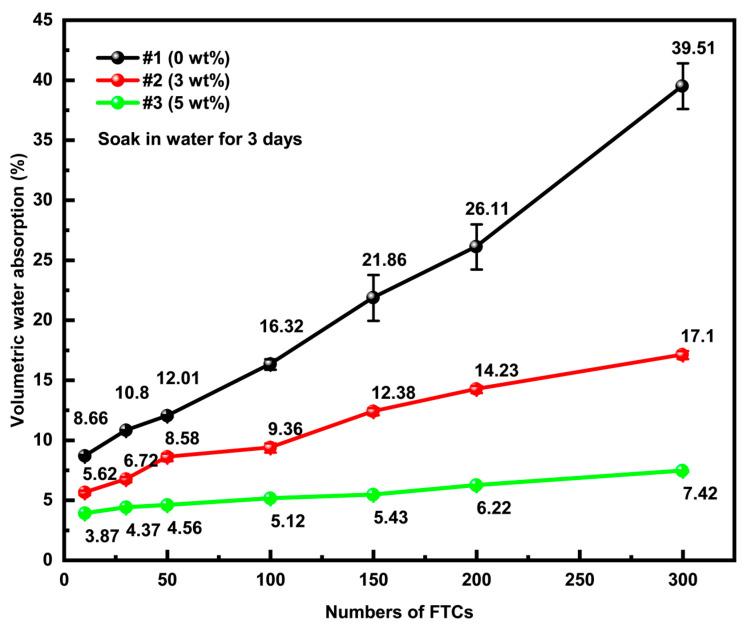
Volumetric water absorption rate of ACEPS after FTCs.

Figure 4 illustrates the trend of volumetric water absorption in specimens with varying aerogel contents under the influence of FTCs. The volumetric water absorption of all specimens increased as the number of FTCs increased, although the rate of increase gradually slowed with further cycles. Specifically, for specimens with 0 wt% aerogel, the volumetric water absorption was 8.66%, 10.80%, 12.01%, 16.32%, 21.86%, 26.11%, and 39.51% after 10, 30, 50, 100, 150, 200, and 300 FTCs, respectively. In comparison, specimens containing 3 wt% aerogel exhibited significantly lower volumetric water absorption values of 5.62%, 6.72%, 8.58%, 9.36%, 12.38%, 14.23%, and 17.10%, respectively. For specimens with 5 wt% aerogel, the volumetric water absorption was further reduced, with values of 3.87%, 4.37%, 4.56%, 5.12%, 5.43%, 6.22%, and 7.42%, respectively. These results clearly indicate that aerogel incorporation significantly reduces the volumetric water absorption of ACEPS, effectively mitigating the negative impact of FTCs on water absorption properties. Notably, for the 5 wt% aerogel specimens, the volumetric water absorption remained below 8% even after 300 FTCs, demonstrating excellent resistance to freeze–thaw degradation.

Additionally, a noticeable difference in volumetric water absorption was observed between specimens treated with FTCs and those in the natural state. Although both sets of specimens exhibited increased water absorption over time, the increase was significantly higher in specimens subjected to FTCs. Notably, the volumetric water absorption of specimens in the natural state reached near saturation after 3 days of immersion, indicating minimal damage during the immersion process. However, after only 10 FTCs, the volumetric water absorption of the specimens increased to 8.66% (0 wt%), 5.62% (3 wt%), and 3.87% (5 wt%), which was significantly higher than their corresponding values in the natural state. This result further underscores the pronounced effect of FTCs on volumetric water absorption, highlighting the accelerated degradation of water absorption properties in response to freeze–thaw cycling.

### 3.3. Mass Change and Appearance Observation

**Figure 5:**
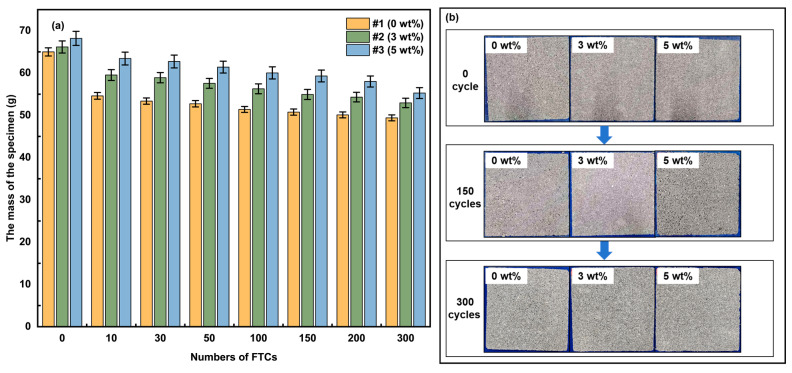
(**a**) Map of ACEPS quality changes; (**b**) surface morphology of ACEPS before and after FTCs.

Figure 5a illustrates the mass loss of specimens with varying aerogel contents subjected to different numbers of FTCs. Aerogel is introduced as an additive (calculated as wt% of the total mass) rather than as a partial replacement for cement to enhance the functional properties of the material while maintaining a constant cement content. This approach ensures the consistency of the baseline matrix composition. The experimental data reveal that mass loss increased progressively with the number of FTCs for all specimens. However, the incorporation of aerogel significantly mitigated the extent of mass loss. Specifically, after 10, 150, and 300 FTCs, the mass loss was 1.6%, 2.2%, and 2.4% for the 0 wt% specimens, 1.0%, 1.7%, and 2.0% for the 3 wt% specimens, and 0.7%, 1.3%, and 1.9% for the 5 wt% specimens, respectively. The mass loss in ACEPS during FTCs is primarily attributed to the spalling of surface material and the drainage of pore water. When moisture infiltrates the material and undergoes a phase transition, the volumetric expansion of ice crystals induces stress, leading to the formation and propagation of microcracks. As the number of FTCs increases, these cracks can evolve, causing material degradation, particle detachment, and consequent mass loss.

The mass loss behavior of ACEPS during FTCs is, thus, governed by both surface material spalling and pore water drainage. When moisture penetrates and undergoes the ice-water phase transition, the expansion of ice crystals generates internal stresses, triggering the formation and growth of microcracks. As the FTC count increases, these cracks may progress, ultimately leading to structural deterioration and the detachment of particles or matrix, resulting in a loss of quality. The specimens with higher aerogel content, such as specimen #3 (5 wt%), consistently exhibited lower mass loss compared to specimens #2 (3 wt%) and #1 (0 wt%). This can be attributed to the superior hydrophobic properties of the aerogel, which effectively reduce water absorption in ACEPS, thereby mitigating internal stresses caused by the water–ice phase transition during FTCs. Additionally, the porous structure of aerogel helps buffer the stress induced by ice crystal expansion, inhibiting the formation and growth of microcracks. These combined properties enable specimens with higher aerogel content to exhibit enhanced freeze–thaw resistance.

While the addition of aerogel significantly improved the freeze–thaw resistance of ACEPS, all specimens still experienced some degree of mass loss as the number of FTCs increased. This indicates that the durability of the material remains challenged under prolonged freeze–thaw conditions. Figure 5b presents a comparison of the specimens’ morphology before and after 300 FTCs. No visible cracking or spalling was observed in any specimen after 300 FTCs, relative to their condition before the test, indicating that the microstructural damage did not reach the critical threshold for initiating macroscopic cracking. This suggests that while the material experiences some degradation during FTCs, the damage remains within a manageable range at this stage.

### 3.4. Compressive Strength

**Figure 6:**
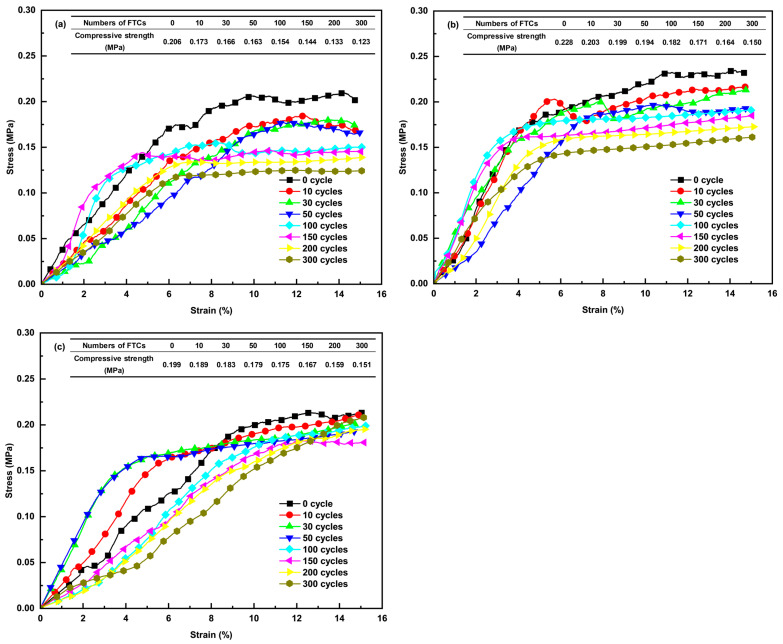
Stress–strain curves of ACEPS after exposure to FTCs: (**a**) specimen #1 (0 wt%); (**b**) specimen #2 (3 wt%); and (**c**) specimen #3 (5 wt%).

The stress–strain curves of ACEPS with varying aerogel contents are presented in Figure 6. Results indicate that incorporating 3 and 5 wt% aerogel has no significant influence on specimen compressive strength. The compressive strengths of specimens #1 (0 wt%), #2 (3 wt%), and #3 (5 wt%) were 0.206, 0.228, and 0.199 MPa, respectively, in their natural state. As an inorganic amorphous material with high porosity, low density, and hydrophobicity, aerogel demonstrates excellent dispersibility in cement-based composite systems. The incorporation of 3 and 5 wt% aerogel does not reach the critical content needed to disrupt the cement paste matrix skeleton. At these levels, aerogel particles remain limited and uniformly distributed throughout the cement matrix, minimally influencing hydration product formation and pore structure connectivity. Although aerogel has relatively weak mechanical properties, its low mass fraction neither enhances nor undermines the load-bearing framework of the cementitious matrix.

The results indicate that the compressive strength of the sample gradually decreases with increasing number of FTCs. For specimen #1 (0 wt%), the compressive strengths were 0.206, 0.173, 0.166, 0.163, 0.154, 0.144, 0.133, and 0.123 MPa at 0, 10, 30, 50, 100, 150, 200, and 300 FTCs, respectively. Specimens #2 (3 wt%) and #3 (5 wt%) exhibited a similar trend of decreasing compressive strength. After 300 FTCs, the compressive strength reductions for the specimens with three different aerogel contents were 40%, 34%, and 24%, respectively. This behavior is primarily attributed to the structural damage caused by the freezing and expansion of moisture inside the specimens during FTCs. As the temperature decreases, the moisture within the material freezes, and the volumetric expansion of the ice damages the structure of the material, resulting in a decrease in compressive strength. Therefore, it can be concluded that FTCs significantly impact the compressive strength of ACEPS, and the inclusion of hydrophobic aerogel effectively mitigates the detrimental effects of FTCs on the compressive strength of the material.

To quantitatively characterize the relationship between the compressive strength of ACEPS after FTCs and the number of FTCs, the experimental results were curve-fitted using Equation (3):

(3) $$Z(N_{Indoor})=B_{1}+A_{1}e^{\left(\frac{-N_{Indoor}}{\beta }\right)}$$

where *Z* represents the compressive strength of the corresponding FTCs, and *B*_1_, *β*, and *A*_1_ are fitting parameters, with *B*_1_ + *A*_1_ = *Z*(0). The fitting analysis yielded coefficients of determination, R^2^, which was greater than 0.95 for all cases, indicating a high degree of confidence in the fitting results and a significant correlation between compressive strength and the number of FTCs. The parameters *B*_1_, *β*, and *A*_1_ obtained from the fitting process are presented in Table 1.

### 3.5. Thermal Conductivity

**Figure 7:**
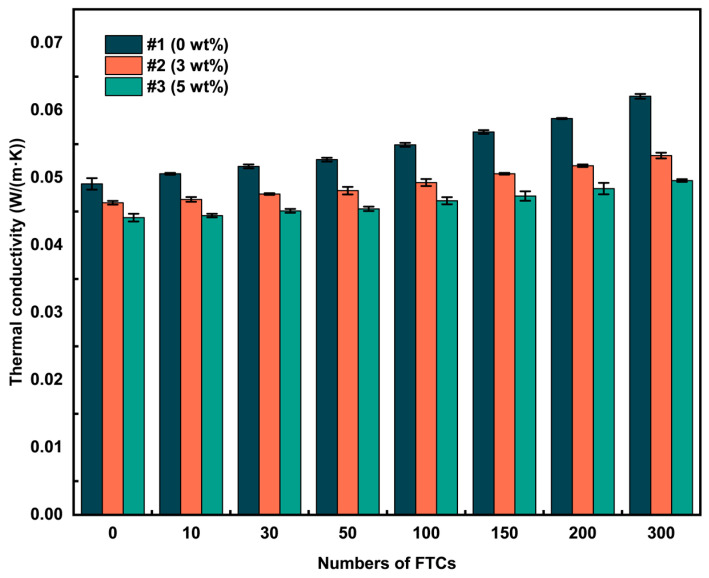
Thermal conductivity of ACEPS after FTCs.

Figure 7 illustrates the trend in the thermal conductivity of ACEPS after undergoing FTCs. The initial thermal conductivities of the specimens were 0.0491, 0.0463, and 0.0441 W/(m·K) for specimens #1 (0 wt%), #2 (3 wt%), and #3 (5 wt%), respectively. After 300 FTCs, the thermal conductivities increased to 0.0621, 0.0533, and 0.0496 W/(m·K), corresponding to increases of 0.0130, 0.0070, and 0.0055 W/(m·K), respectively. In a similar freeze–thaw environment, Li et al. [25] found that the polyphenol insulation board’s thermal conductivity increased by 0.0137 W/(m·K) after 50 FTCs in freeze–thaw testing. This result indicates that the rate at which the thermal conductivity of ACEPS increases is lower than that of polyphenol insulation in freeze–thaw environments, demonstrating the better insulation performance of the former in cold regions. These results indicate that the incorporation of aerogel significantly mitigates the increase in thermal conductivity despite the negative effects of FTCs on ACEPS. This can primarily be attributed to the water absorption characteristics of the specimens. The excellent hydrophobicity of the aerogel effectively reduces water absorption, thereby slowing the intrusion of water and freezing during the freeze–thaw process, which, in turn, helps preserve the microstructure and minimizes the deterioration of thermal conductivity.

To further investigate the relationship between thermal conductivity and the number of FTCs, the experimental data were analyzed through linear fitting using Equation (4). The fitting results show a significant linear positive correlation between the thermal conductivity of ACEPS and the number of FTCs, with correlation coefficients (R^2^) all greater than 0.99, indicating the high reliability of the fitting results. The parameters *a* and *b* for the fitted specimens #1 (0 wt%), #2 (3 wt%), and #3 (5 wt%) are provided in Table 2. Equation (4) shows the relationship between the thermal conductivity of the material and the number of indoor FTCs, indicating a progressive increase in thermal conductivity with each additional cycle. In particular, the thermal conductivity increases by a constant factor (*a*) for every single FTC.

(4) $$y(N_{Indoor})=aN_{Indoor}+b$$

where *y* represents the thermal conductivity of the corresponding FTC; *a* represents the fitting parameter, and *b* represents the initial thermal conductivities of the specimens.

### 3.6. Pore Structure

**Figure 8:**
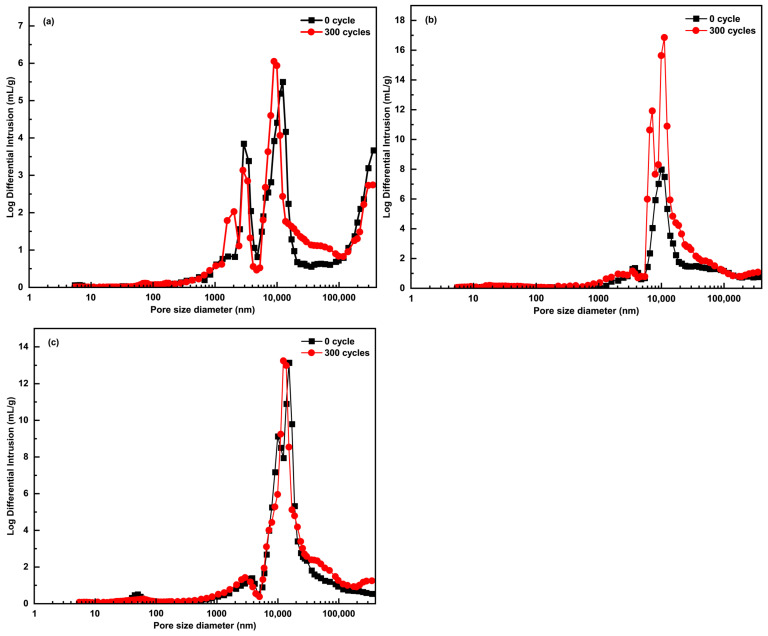
Approximate macropore size distribution from the mercury intrusion branch for ACEPS: (**a**) specimen #1 (0 wt%); (**b**) specimen #2 (3 wt%); (**c**) specimen #3 (5 wt%).

As shown in Figure 8, the pore volume of ACEPS increases gradually with the addition of aerogel, while specimens with aerogel exhibit a more concentrated pore size distribution. This indicates that the introduction of aerogel significantly optimizes the pore size distribution of ACEPS. Further analysis revealed that after 300 FTCs, the pore size distribution of each specimen changed to varying extents. Notably, specimen #1 (0 wt%) showed a more significant variation in pore size within the range of 20,000 to 100,000 nm, while the pore size distribution of specimen #2 (3 wt%) was more concentrated around 10,000 nm. Specimen #3 (5 wt%) exhibited relatively small changes in its pore size distribution. As shown in Table 3, aerogel doping notably influenced the porosity and average pore size of the material. The porosity of specimen #1 (0 wt%) was 61.5%, with an average pore size of 1688.89 nm; in contrast, specimen #2 (3 wt%) exhibited a significant increase in porosity to 83.3%, with a reduced average pore size of 964.08 nm. In specimen #3 (5 wt%), the porosity increased further to 90.6%, while the average pore size slightly decreased to 883.92 nm. This suggests that aerogel not only significantly increases the porosity of the material but also refines its pore size distribution. After 300 FTCs, the porosity and average pore size of all specimens increased, although the magnitude of the changes varied. Specifically, the porosity of specimen #1 (0 wt%) increased from 61.5% to 68.4%, and the average pore size increased significantly from 1688.89 to 3239.88 nm, indicating greater damage to its pore structure due to FTCs. In comparison, the porosity of specimen #2 (3 wt%) increased from 83.3% to 89.9%, and the average pore size increased from 964.08 to 1556.14 nm. Meanwhile, the porosity of specimen #3 (5 wt%) increased from 90.6% to 94.2%, and the average pore size slightly increased from 883.92 to 1085.30 nm. The relatively small changes in porosity and average pore size for specimens #2 (3 wt%) and #3 (5 wt%) suggest that the addition of aerogel helped suppress the deterioration of the pore structure caused by FTCs.

In this study, the aerogel used contains methyl groups, which impart excellent hydrophobic properties. These hydrophobic characteristics effectively prevent water penetration and minimize water adsorption within the composite structure, thereby significantly reducing the amount of residual water that could expand during freezing. This feature directly enhances the material’s resistance to freeze–thaw damage. As shown in Figure 8c, our experimental results demonstrate that the material maintains stable performance even after prolonged FTCs. This finding further validates the positive role of the mesoporous structure introduced by hydrophobic aerogel in improving the long-term stability of the ACEPS composite.

**Figure 9:**
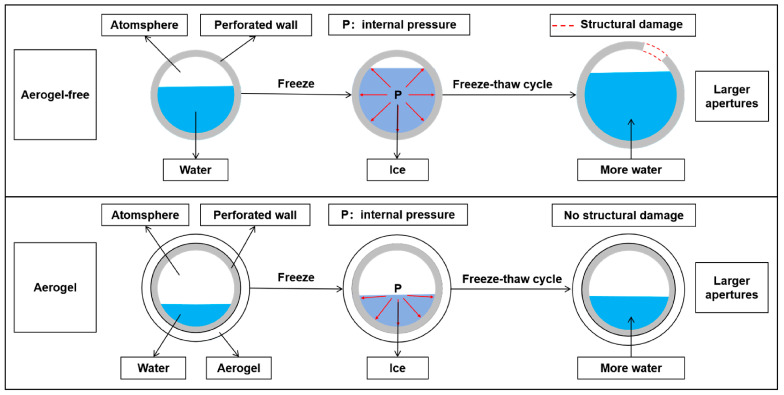
Diagram of the freeze–thaw damage mechanism.

The mechanism of damage inhibition of ACEPS by hydrophobic aerogels during FTCs is illustrated in Figure 9. During FTCs, water in the pores of the material undergoes a phase transition from liquid to solid (ice), resulting in volume expansion and a significant increase in internal pore pressure, which can cause the pore structure to expand or even rupture. This destruction of the pore structure not only reduces the compressive strength of the material but also increases thermal conductivity by shortening the heat transfer path. As FTCs progress, more moisture penetrates the pore structure, further accumulating internal pressure and exacerbating the freeze–thaw damage to ACEPS. However, with the introduction of hydrophobic aerogel into the ACEPS system, the aerogel primarily distributes around the pores. With its excellent hydrophobic properties, the aerogel effectively prevents water from entering the pores, significantly reducing the internal pressure generated during the liquid-to-solid phase transition. This reduction in pressure slows the damage caused by FTCs to both the microstructure and macroscopic properties of the material. The experimental results indicated that the porosity of specimens #1 (0 wt%), #2 (3 wt%), and #3 (5 wt%) increased under FTCs, along with a corresponding increase in average pore size. This phenomenon is primarily attributed to the water phase transition and accompanying volume expansion, which disrupts the internal structure of ACEPS and creates larger pores. However, the extent of pore expansion was closely linked to the aerogel content. Specifically, the pore structure of specimen #1 (0 wt%), which contained no aerogel, was more susceptible to damage from FTCs, leading to a significant increase in average pore size. In contrast, the increase in pore size for specimen #2 (3 wt%) was much smaller, and the increase for specimen #3 (5 wt%) was even smaller than that of specimen #2 (3 wt%). This trend indicates that incorporating up to 5 wt% of aerogel effectively optimizes the pore structure and minimizes the formation of large pores. Additionally, the high specific surface area and hydrophobic properties of the aerogel significantly reduced the water absorption of ACEPS, thereby decreasing the amount of freezable water in the pore structure and further inhibiting freeze–thaw damage.

### 3.7. Prediction of Service Life Under Freeze–Thaw Conditions

#### 3.7.1. Experimental Life Prediction Model

The structural experimental lifetime of ACEPS under FTCs in laboratory conditions can be further derived using Equation (5):

(5) $$N_{Indoor}=-\beta \ln\left|\frac{Z(N_{Indoor})-B_{1}}{A_{1}}\right|$$

Similarly, the expression for the functional experimental lifetime of ACEPS under laboratory conditions can be obtained from Equation (6) as follows:

(6) $$N_{Indoor}=\frac{\left(y(N_{Indoor})-b\right)}{a}$$

#### 3.7.2. ACEPS Rapid Freeze–Thaw Test Lifetime

According to the requirements of Chinese specification JG/T 536-2017 [48], the compressive strength of the specimen must be at least 0.15 MPa. The structural experimental lifetime of ACEPS with three different aerogel contents under rapid freeze–thaw test conditions can be calculated using Equation (5). When the thermal conductivity requirements are not higher than 0.050, 0.055, and 0.060 W/(m·K), respectively, the functional experimental lifetimes of ACEPS with three different aerogel contents under rapid freeze–thaw test conditions can be further determined based on Equation (6).

As shown in Table 4, the structural experimental lifetime of specimen #1 (0 wt%) was 69.6 rapid FTCs, while its functional experimental lifetimes (evaluated by thermal conductivity values of 0.050, 0.055, and 0.060 W/(m·K), respectively) were 19.0, 124.8, and 230.6 rapid FTCs. In contrast, the structural experimental lifetime of specimen #2 (3 wt%) was significantly improved to 209.3 rapid FTCs, with functional experimental lifetimes of 143.5, 337.5, and 531.5 rapid FTCs, respectively. For specimen #3 (5 wt%), the structural experimental lifetime was 548.4 rapid FTCs, while the functional experimental lifetime was further improved to 293.4, 542.1, and 790.7 rapid FTCs, respectively. The results demonstrate that the introduction of aerogel significantly enhances the freeze–thaw experimental lifetime of ACEPS compared to CEPS without aerogel doping. The addition of aerogel not only improves the compressive properties of the material but also effectively delays the degradation of thermal and mechanical properties during FTCs by optimizing the pore structure and reducing thermal conductivity. In particular, the high-content aerogel specimen (#3 (5 wt%)) exhibited excellent freeze–thaw resistance, with both #3 (5 wt%) and the low-content aerogel specimen (#2 (3 wt%)) showing significantly superior structural experimental lifetimes compared to the non-aerogel specimen (#1 (0 wt%)). Moreover, its functional experimental lifetime was notably better than both the low-content aerogel specimen (#2 (3 wt%)) and the non-aerogel specimen (#1 (0 wt%)).

#### 3.7.3. Actual Service Life of FTCs Under Natural Environmental Conditions

**Figure 10:**
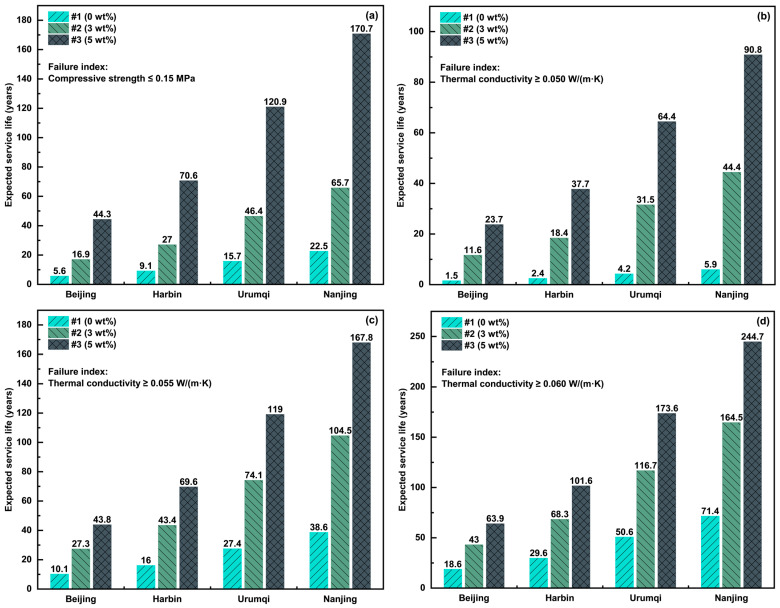

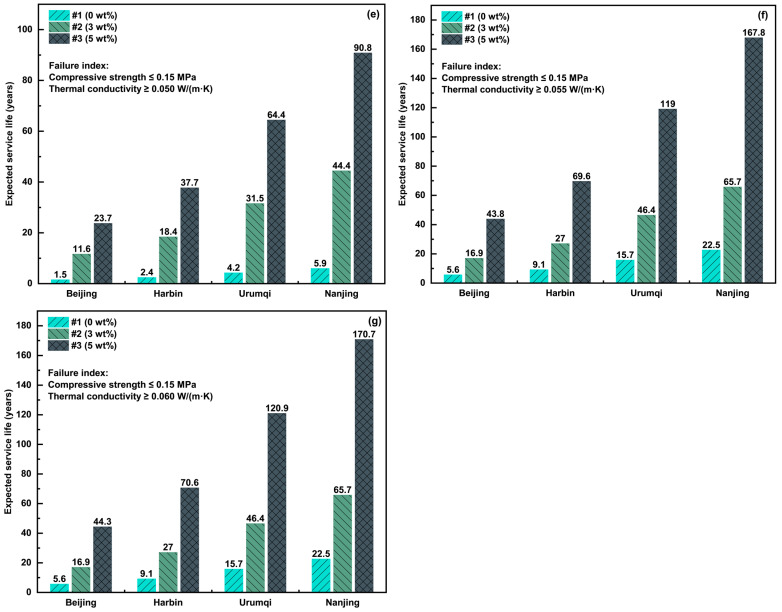
Predicted structural and functional service life of ACEPS: (**a**–**d**) single-index and (**e**–**g**) two indices.

In this study, temperature data from four representative regions in China (Beijing, Harbin, Urumqi, and Nanjing) were collected over the past five years (2020–2024). The process of temperature changing from positive to negative and back to positive, or from negative to positive and back to negative, was defined as a single FTC. Statistical analysis of the temperature data revealed that Beijing, Harbin, Urumqi, and Nanjing experienced 402, 253, 148, and 105 FTCs, respectively, over the past five years. The single failure criteria for compressive strength and thermal conductivity were further predicted using Equation (2), and the experimental life prediction model for the service life of ACEPS under natural environmental conditions in the four regions was applied. The actual service life assessment system for ACEPS was constructed by coupling the mechanical properties of the material with its thermal insulation function. When the compressive strength or thermal conductivity of the material exceeds a preset critical threshold, the material is deemed to have failed in service. Based on single and two-parameter failure criteria, the actual FTC service life of ACEPS under natural environmental conditions in the different regions was predicted. The results are shown in Figure 10.

The life prediction results reveal that the frequency of FTCs in different regions significantly impacts the actual service life of ACEPS. The structural and functional lifetimes of the specimens were the shortest in Beijing, which experiences the highest average annual number of FTCs, while those in Nanjing were the longest, owing to the lowest average annual FTCs. Specimen #1 (0 wt%), which lacked aerogel doping, consistently exhibited the shortest structural lifetime across all regions. In contrast, the structural lifetime of specimens #2 (3 wt%) and #3 (5 wt%) increased significantly with higher aerogel content, with specimen #3 (5 wt%) (high aerogel content) showing the best structural performance in all regions. The functional lifetime also improved significantly with increased aerogel dosage, and the more relaxed thermal conductivity requirements (i.e., the higher the thermal conductivity threshold), the longer the functional lifetime. For example, in Harbin, when the thermal conductivity requirement was 0.050 W/(m·K), the functional lifetime of specimen #1 (0 wt%) was 2.4 years, while that of specimen #3 (5 wt%) reached 37.7 years. When the thermal conductivity requirement was relaxed to 0.060 W/(m·K), the functional lifetime of specimen #3 (5 wt%) further increased to 101.6 years. This highlights the significant role of aerogel in enhancing functional lifetime, particularly in colder regions.

In the combined prediction based on the dual failure metrics, the overall service life of specimens #2 (3 wt%) and #3 (5 wt%) was notably superior to that of specimen #1 (0 wt%). For specimen #3 (5 wt%) (high aerogel content), the actual service life was primarily governed by structural failure. For specimen #2 (3 wt%) (medium aerogel content), structural failure dominated when the thermal conductivity thresholds were 0.055 and 0.060 W/(m·K). However, under the more stringent thermal conductivity requirement (0.050 W/(m·K)), functional failure became the primary limiting factor. In contrast, the actual service life of specimen #2 (3 wt%) (no aerogel) was dominated by functional failure. The introduction of aerogel significantly improves the freeze–thaw resistance of ACEPS, effectively decelerating the degradation process, particularly in colder regions such as Harbin and Urumqi.

#### 3.7.4. Life-Cycle Cost and Scale Production Feasibility Analysis

The previous analysis yielded the service lifetimes of #1 (0 wt%), #2 (3 wt%), and #3 (5 wt%) under different requirement conditions. With the continuous improvement in building energy efficiency standards, building exterior wall insulation materials with low thermal conductivity have become the preferred choice. Therefore, in this study, we selected the constraint conditions (indicated in Figure 10e), with a compressive strength index of 0.15 MPa and thermal conductivity of 0.050 W/(m·K), as the basis for analysis. According to engineering economics theory, the whole life-cycle cost of buildings can be evaluated through annual cost A (unit: CNY/m^3^), calculated as

(7) $$A=P\frac{i{\left(1+i\right)}^{n}}{{\left(1+i\right)}^{n}-1}$$

where P is the initial material cost; n is the service life cycle, and i is the discount rate.

The current cost of aerogel is 70 CNY/kg. In this study, the initial cost of #1 (0 wt%) was set to 300 CNY/m^3^; #2 (3 wt%) and #3 (5 wt%), based on the proportion of #1 (0 wt%), were incorporated with 3 and 5 wt% of aerogel, respectively, which caused the initial costs to increase to 510 and 650 CNY/m^3^, respectively. According to the analysis results in Figure 10e, the service lifetimes of #1 (0 wt%), #2 (3 wt%), and #3 (5 wt%) in Beijing, Harbin, Urumqi, and Nanjing are as follows:

#1 (0 wt%): Service life in Beijing, Harbin, Urumqi, and Nanjing is 1.5, 2.4, 4.2, and 5.9 years, respectively;

#2 (3 wt%): Service life in Beijing, Harbin, Urumqi, and Nanjing is 11.6, 18.4, 31.5, and 44.4 years, respectively;

#3 (5 wt%): Service life in Beijing, Harbin, Urumqi, and Nanjing is 23.7, 37.7, 64.4, and 90.8 years, respectively.

Because the incremental cost of near-zero energy buildings transfers to consumers through sales prices, using the real estate industry’s discount rate for economic evaluation is inappropriate. Instead, referencing China’s current social discount rate is more suitable. The “Construction Project Economic Evaluation Methods and Parameters (Third Edition)” indicates that the social discount rate is generally 8%, with possible reductions for projects with greater long-term benefits. As low-energy buildings are projects whose benefits manifest over a longer period, this study adopts 6% as the social discount rate.

The annual costs for #1 (0 wt%), #2 (3 wt%), and #3 (5 wt%) were calculated using Formula (7) (see Table 5). Results show that the life-cycle cost of #1 (0 wt%) in Beijing is 210.2 CNY/m^3^, while #3 (5 wt%), despite its higher initial costs than that of #1 (0 wt%), exhibits reduced life-cycle costs (52.1 CNY/m^3^) in Beijing. In the Urumqi and Nanjing regions, the life-cycle costs of #2 (3 wt%) are lower than those of #3 (5 wt%), indicating the greater economic viability of the former.

The technology proposed in this study is feasible for large-scale application, which is mainly reflected in two aspects: First, the material offers significant life-cycle cost advantages, effectively reducing the long-term economic burden of construction projects. Second, its production involves standardized equipment and mature processes, indicating that it can be easily scaled, fully meeting the technical requirements for mass production.

## 4. Conclusions

This study systematically examined the effects of hydrophobic aerogel on key performance indicators of ACEPS, including compressive strength, volumetric water absorption, thermal conductivity, and porosity under FTC conditions. The freeze–thaw resistance of ACEPS was explored, and the actual service life of ACEPS under FTCs in four regions—Beijing, Harbin, Urumqi, and Nanjing—was evaluated through a life prediction model. The main findings and their implications are summarized as follows:

(1) In its natural state, the volumetric water absorption of ACEPS gradually increased with soaking time, stabilizing on the third day at maximum saturation. The incorporation of hydrophobic aerogel significantly reduced the volumetric water absorption of ACEPS, highlighting its role in limiting water intrusion. This finding has significant implications for moisture management in building envelopes, particularly in regions with high precipitation or humidity, where reduced water absorption can substantially mitigate moisture-related deterioration mechanisms and enhance the long-term durability of insulation systems;
(2) The compressive strength of ACEPS gradually decreased with the number of FTCs. ACEPS with hydrophobic aerogel demonstrated a slower rate of compressive strength degradation compared to the aerogel-free specimens. After undergoing FTCs, the maximum compressive strength decay reached 40% in the aerogel-free condition, whereas the aerogel-doped condition significantly slowed this degradation process. This enhanced mechanical stability has direct practical applications in load-bearing insulation systems, where maintaining structural integrity under cyclic environmental stresses is critical for building safety and longevity. The improved performance could potentially extend maintenance intervals and reduce the life-cycle costs of building envelope systems;
(3) The thermal conductivity of ACEPS showed a gradual increase with the number of FTCs. After 300 FTCs, the thermal conductivity of specimens with 0 wt%, 3 wt%, and 5 wt% aerogel increased by 0.0130, 0.0070, and 0.0055 W/(m·K), respectively. This substantial improvement in thermal stability has profound implications for energy conservation in buildings. By maintaining lower thermal conductivity values over time, aerogel-modified ACEPS can significantly reduce heating energy consumption in cold regions, potentially decreasing annual energy costs by 10–15% compared to conventional insulation systems that experience more rapid thermal performance degradation;
(4) The incorporation of aerogel significantly slowed the evolution of the microporous structure of ACEPS under freeze–thaw conditions. The changes in porosity and average pore size of ACEPS after FTCs were significantly reduced with increasing aerogel doping. This microstructural stability translates to enhanced acoustic performance and fire resistance properties, broadening the multifunctional applications of ACEPS in high-performance building envelopes. The preserved microporous structure also suggests potential applications in moisture-buffering and indoor air quality improvement;
(5) The incorporation of aerogel significantly enhanced the structural and functional service life of ACEPS, with the effects becoming more pronounced as the aerogel content increased. The dramatic service life extension—reaching up to 100 years for functional life in some scenarios—represents a paradigm shift in building material durability. This could fundamentally alter construction practices in cold regions, enabling “build once” approaches that minimize reconstruction and renovation needs over building lifespans. The economic implications are substantial, with potential life-cycle cost reductions of 15–20% when accounting for avoided maintenance and replacement costs.

The practical applications of these findings extend beyond residential buildings to critical infrastructure in cold regions, including hospitals, data centers, and industrial facilities, where thermal performance stability is paramount. This technology could be particularly valuable for remote or difficult-to-access installations where maintenance operations are challenging and costly. Furthermore, the enhanced durability aligns with sustainability goals by reducing embodied carbon associated with material replacement and repair.

However, this study has limitations, which can be transformed into directions for future research. The FTC tests were performed under controlled laboratory conditions, whereas real-world freeze–thaw processes are influenced by more complex environmental factors. To bridge the gap between experimental research and practical applications, we recommend demonstration projects in different climatic conditions to validate the laboratory findings in real-world scenarios. Current standards have relatively low requirements for the mechanical properties of the insulation layer. Moreover, shear strength and tensile strength are not included as evaluation criteria for freeze–thaw characteristics of insulation materials. Thus, in this study, we did not measure these properties. Future studies could focus on direct testing of these mechanical parameters to evaluate the overall mechanical performance of aerogel-modified composite insulation materials under cyclic environmental conditions.

## Figures and Tables

**Table 1:** Compressive strength fitting parameters and correlation coefficients.

Sample	*B*_1_	*β*	*A*_1_	R^2^
#1 (0 wt% (control))	0.1343	45.9325	0.0715	0.95
#2 (3 wt%)	0.1553	79.9874	0.0726	0.96
#3 (5 wt%)	0.1495	119.3902	0.0494	0.97

**Table 2:** Fitted parameters and correlation coefficients for thermal conductivity after FTCs.

Sample	*a*	*b*	R^2^
#1 (0 wt% (control))	4.7277 × 10^−5^	0.0491	0.99
#2 (3 wt%)	2.5777 × 10^−5^	0.0463	0.99
#3 (5 wt%)	2.0108 × 10^−5^	0.0441	0.99

**Table 3:** Pore structure of ACEPS.

Specimen Status	Test Items	Sample		
		#1 (0 wt% (Control))	#2 (3 wt%)	#3 (5 wt%)
Pre-freeze–thaw	Porosity (%)	61.5	83.3	90.6
	Average pore size (nm)	1688.89	964.08	883.92
Post-freeze–thaw	Porosity (%)	68.4	89.9	94.2
	Average pore size (nm)	3239.88	1556.14	1085.30

**Table 4:** Prediction of experimental lifetime under indoor FTCs.

Failure Indicators	Sample	Maximum Life (Times)
Compressive strength ≤0.15 MPa	#1 (0 wt% (control))	69.6
	#2 (3 wt%)	209.3
	#3 (5 wt%)	548.4
Thermal conductivity ≥0.050 W/(m·K)	#1 (0 wt% (control))	19.0
	#2 (3 wt%)	143.5
	#3 (5 wt%)	293.4
Thermal conductivity ≥0.055 W/(m·K)	#1 (0 wt% (control))	124.8
	#2 (3 wt%)	337.5
	#3 (5 wt%)	542.1
Thermal conductivity ≥0.060 W/(m·K)	#1 (0 wt% (control))	230.6
	#2 (3 wt%)	531.5
	#3 (5 wt%)	790.7

**Table 5:** Annual Cost of Insulation Material in Different Cities (Unit: CNY/m^3^).

Sample	Beijing	Harbin	Urumqi	Nanjing
#1 (0 wt% (control))	210.2	135.8	83.4	62.0
#2 (3 wt%)	62.3	46.5	36.4	33.1
#3 (5 wt%)	52.1	43.9	39.9	39.2

## Data Availability

The original contributions presented in this study are included in the article/Supplementary Materials. Further inquiries can be directed to the corresponding author.

## Supplementary Materials

The following supporting information can be downloaded at https://www.mdpi.com/article/10.3390/ma18112646/s1, Table S1: Test conditions.

## Abbreviations

The following abbreviations are used in this manuscript:

ACEPS board	Aerogel cement-based EPS insulation board
EPS	Expanded polystyrene
FTCs	Freeze–thaw cycles
CEPS board	Non-combustible expanded polystyrene board
LD	Linear dichroism
