# Supplementary material for: Study on Freeze–Thaw Cycle Performance and Regional Service Life Prediction of Hydrophobic Aerogel-Modified ACEPS Boards
Source: Materials (Basel). 2025 Jun 5;18(11):2646. doi: 10.3390/ma18112646 (PMC12155739; doi:10.3390/ma18112646)
Supplement: Supplementary file 1 [file materials-18-02646-s001.zip › materials-3660401-supplementary.pdf]

Supplementary Materials

Table S1. Test conditions.

Operating condition	Test Programs	Numbers of FTCs	Test specimen size (mm)
1#: Aerogel content 0 wt%	Volumetric water absorption	0, 10, 30, 50, 100, 150, 200, 300	100 × 100 × 50
	Compressive strength	0, 10, 30, 50, 100, 150, 200, 300	100 × 100 × 50
	Thermal conductivity	0, 10, 30, 50, 100, 150, 200, 300	300 × 300 × 30
	Pore structure	0, 300	10 × 10 × 10
2#: Aerogel content 3 wt%	Volumetric water absorption	0, 10, 30, 50, 100, 150, 200, 300	100 × 100 × 50
	Compressive strength	0, 10, 30, 50, 100, 150, 200, 300	100 × 100 × 50
	Thermal conductivity	0, 10, 30, 50, 100, 150, 200, 300	300 × 300 × 30
	Pore structure	0, 300	10 × 10 × 10
3#: Aerogel content 5 wt%	Volumetric water absorption	0, 10, 30, 50, 100, 150, 200, 300	100 × 100 × 50
	Compressive strength	0, 10, 30, 50, 100, 150, 200, 300	100 × 100 × 50
	Thermal conductivity	0, 10, 30, 50, 100, 150, 200, 300	300 × 300 × 30
	Pore structure	0, 300	10 × 10 × 10
